# WISP1-αvβ3 integrin signaling positively regulates TLR-triggered inflammation response in sepsis induced lung injury

**DOI:** 10.1038/srep28841

**Published:** 2016-06-28

**Authors:** Zhixia Chen, Xibing Ding, Shuqing Jin, Bruce Pitt, Liming Zhang, Timothy Billiar, Quan Li

**Affiliations:** 1 Department of Anesthesiology, East Hospital, Tongji University school of Medicine, Shanghai, China; 2 Department of Surgery, University of Pittsburgh Medical Center, Pittsburgh, Pennsylvania, United States of America; 3 Department of Environmental and Occupational Health, Graduate School of Public Health, University of Pittsburgh, Pittsburgh, Pennsylvania, United States of America; 4 Department of Anesthesiology, University of Pittsburgh Medical Center, Pittsburgh, Pennsylvania, United States of America

## Abstract

We recently noted that the matricellular protein WISP1 contributes to sepsis induced acute lung injury (ALI) via integrin β6. In the current study, we pursued further aspects of WISP1 modulation of TLR signaling in lungs of mice after sepsis and TLR4 mediated release of TNF-α in macrophages. After confirming that TLR4 and CD14 are critical in transducing sepsis mediated ALI, we now demonstrate that intrapulmonary αvβ3 is increased by polymicrobrial sepsis in a TLR4, CD14 dependent fashion. Comparison of cultured macrophages revealed that WISP1 increased release of TNF-α from RAW264.7 cells with baseline expression of αvβ3, but primary cultures of peritoneal macrophages (PMø) required activation of TLR4 to induce de novo synthesis of αvβ3 enabling WISP1 to stimulate release of TNF-α. The specific requirement for β3 integrin was apparent when the effect of WISP1 was lost in PMø isolated from β3^−/−^ mice. WISP1 enhanced TLR4 mediated ERK signaling and U0126 (an ERK inhibitor) blocked LPS induced β3 integrin expression and WISP1 enhanced TNF-α release. Collectively these data suggest that WISP1-αvβ3 integrin signaling is involved in TLR4 pathways in macrophages and may be an important contributor to TLR4/CD14 mediated inflammation in sepsis induced lung injury.

Acute lung injury (ALI), is one of the major challenges in clinical practice and responsible for a high degree of morbidity and mortality amongst intensive care patients^1^. A variety of stimuli can initiate ALI, such as severe infection, ischemia/reperfusion, or mechanical ventilation^2^. Sepsis is one of the most important causes of ALI. It is well documented that macrophage activation in response to pathogens and/or tissue damage occurs, at least in part, through TLR signaling pathways that are associated with pro-inflammatory cytokine up-regulation and release of mediators such as TNF-α. In spite of recent clinical and basic science advances in the understanding of the molecular mechanisms of sepsis, mortality rates for ALI remain high^3^,^4^.

WNT1 inducible secreted protein 1 (WISP1 or CCN4; also referred to as WNT1 inducible signaling protein-1) is a secreted, matricellular protein, allocated to the CCN protein family and is involved in cell adhesion, migration, differentiation, proliferation, and survival^5^. Its functions are perhaps best understood in the context of bone turnover as recently described in a genetically engineered whole body WISP1 null mouse^6^. In the lung, WISP1 was first noted to induce hyperplasia and proliferation of alveolar epithelial cells accompanied by an increased expression of matrix metalloproteinases in bleomycin induced fibrosis in mice^7^,^8^. By using an unbiased genomic approach of haplotype association mapping, we demonstrated that WISP1 contributes to the sensitivity of intact mice to high tidal volume ventilation via a TLR4 dependent pathway^9^. Most recently, we reported that WISP1 contributed to polymicrobial sepsis model of ALI in intact mice in part via WISP1-integrin β6 pathway^10^.

Integrins are a family of transmembrane adhesion receptors containing nineteen α subunits and eight β subunits that interact to form up 25 different heterodimers in mammals^11^. By using integrin subunit knockout mice and antibodies, Sheppard D *et al*. found that the αvβ6 integrin on epithelial cells and the αvβ5 integrin on endothelial cells in mediating increases in alveolar permeability in multiple models of acute lung injury^12^. Integrins are also important receptors for matricellular proteins and at least eight integrins (αvβ3, α2β1, α5β1, α6β1, αvβ5, αIIbβ3, αMβ2 and αDβ2) have been identified as signaling receptors mediating various CCN functions^13^. Although we^10^ reported that WISP1-β6 complexes in lung were important in cecal ligation and puncture (CLP), the non-specificity of inhibition of integrins with RGD and the partial effect observed with neutralizing antibodies to β6 suggest that other RGD sensitive integrins may play a role. Less is known about β3 and WISP1, we recently found that WISP1-integrinβ3 interaction contributed to mechanical ventilation augmented PolyI:C induced lung injury^14^. αvβ3 integrin (vitronectin receptor, CD51/CD61) is a ubiquitous receptor that is expressed on a wide variety of cell types including differentiated macrophage^15^,^16^. Although β3 integrin appeared important in endothelial cell barrier protection in intraperitoneal LPS and CLP^17^, other studies have indicated that αvβ3 is linked to inflammation and could be a potential macrophage activator^18^,^19^,^20^,^21^.

In the current study, we noted that: a) intrapulmonary αvβ3 is increased by polymicrobrial sepsis in a TLR4, CD14 dependent fashion; b) β3 is required for WISP1 to enhance TLR4 mediated activation of TNF-α release from primary cultures of PMø; and c) ERK signaling is important in enhancing and transducing WISP1 synergetic effect.

## Results

### Whole body genetic ablation of TLR4 or CD14 reduces the sensitivity of intact mice to cecal ligation and puncture (CLP) induced sepsis

**Figure 1:**
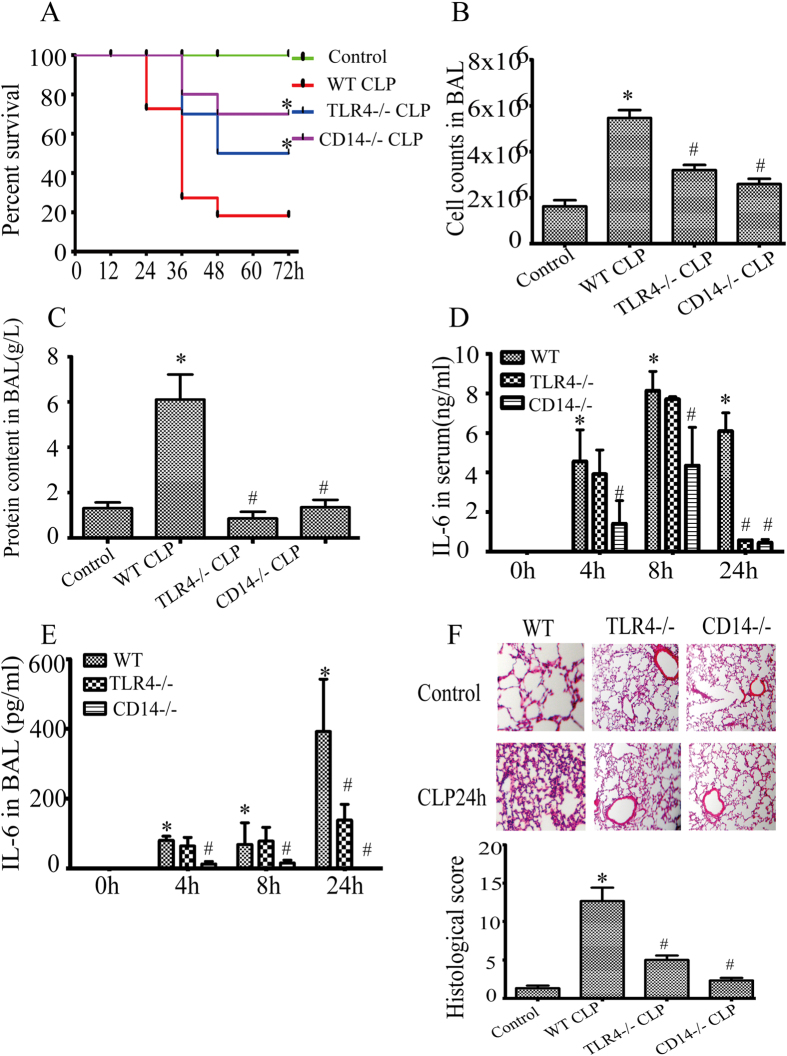
TLR4^−/−^ or CD14^−/−^ reduces the sensitivity to cecal ligation and puncture (CLP) induced sepsis in mice. WT, TLR4^−/−^ or CD14^−/−^ mice were subjected to CLP (n = 10 mice per group) for 72 h to monitor the survival rate, and the Kaplan-Meier method was used to compare the differences between groups. *P < 0.05 versus WT CLP group (**A**). Cell counts (**B**) and total protein content (**C**) in BAL 24 h after CLP. IL-6 levels in the serum (**D**) and BAL (**E**) in mice alive at 0, 4, 8, 24 h were measured by ELISA. Data were expressed as mean ± SEM (n ≥ 5mice per group). *P < 0.05 versus control (0 h), ^#^P < 0.05 versus WT CLP group. (**F**) Lung tissue sections were stained with hematoxylin and eosin. Images(x20) from lung sections were shown (upper panel). Composite lung injury scores represent the sum of the mean injury subtype scores for each condition on a scale of 0–16 (bottom panel). In each section, 5 randomly selected fields were scored for a) interstitial edema b) alveolar edema, c) hemorrhage, and d) neutrophil infiltration are presented on a scale of 0–4 (0 = none,4 = severe). The graph is representative of lung sections from three or more mice per group. *P < 0.05 versus Control, ^#^P < 0.05 versus WT CLP group.

Sepsis is characterized by systemic inflammation, and may lead to end-organ dysfunction. Septic patients are particularly at risk of developing ALI which represents the highest risk factor for mortality^1^. We sought to detect the effect of global genetic ablation of TLR4 or CD14 (TLR4^−/−^, CD14^−/−^) in a clinically relevant model of polymicrobial sepsis caused by CLP in mice. The TLR4/MD2 complex is well characterized as a receptor for Gram-negative bacterial endotoxin^22^,^23^,^24^,^25^ and along with its co-receptor. CD14 is critical for the host response to a variety of infectious and sterile stimuli^26^. As the first step, we noted that cohorts of ten TLR4^−/−^ or CD14^−/−^ mice had significantly higher survival rates at 72 hrs after CLP than their wild type counterparts (Fig. 1A). Moreover, cell counts in the bronchoalveolar lavage fluid (BAL) were significantly less in either TLR4^−/−^ or CD14^−/−^ mice than those in wild type mice at 24 hr after CLP and elevations in protein in BAL and IL-6 in serum or BAL were reduced to almost pre-CLP levels in either TLR4^−/−^ or CD14^−/−^ mice (Fig. 1B–E, respectively). At 24 hr after CLP, histopathology of lungs revealed considerable congestion, interstitial edema and cellular infiltrates in wild type mice, however these were not apparent at the light microscopic level in TLR4^−/−^ or CD14^−/−^ mice. Histopathologic scoring confirmed that the deletion of either TLR4 or CD14 protected mice from acute lung injury (Fig. 1F).

### Ablation of integrin β3 is protective in sepsis induced lung injury

**Figure 2:**
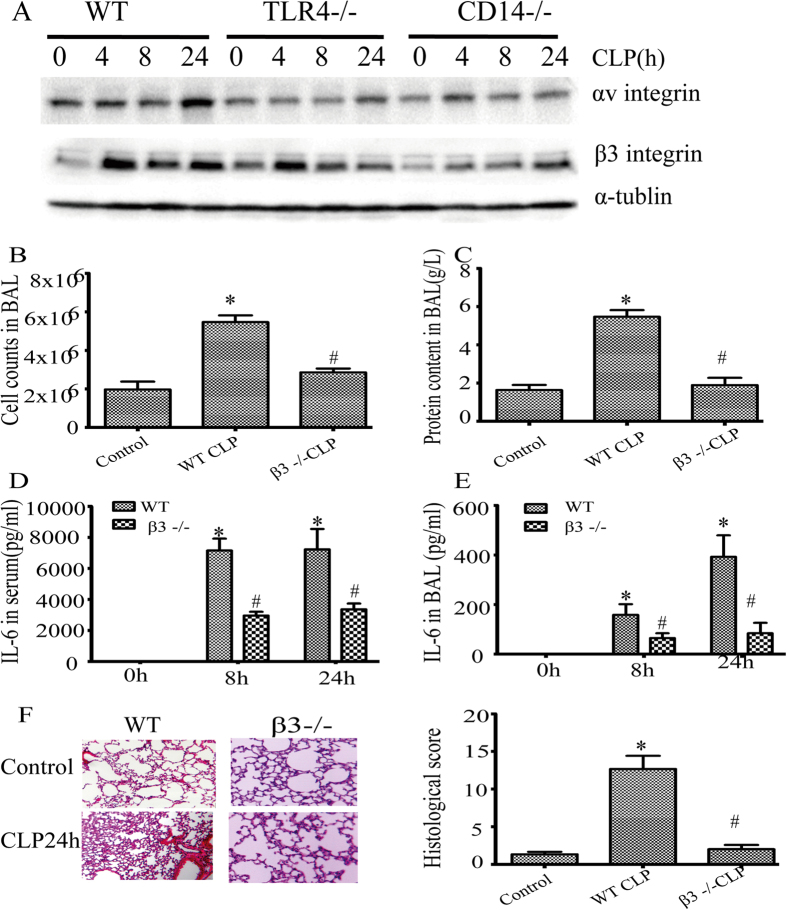
Integrin β3^−^/^−^ is protective in sepsis induced lung injury. WT, TLR4^−/−^, CD14^−/−^ and β3^−/−^ mice were subjected to CLP. (**A**) Cell lysates from lung tissues were probed for αvβ3 integrin expression by western bloting. The blots shown are representative of three experiments with similar results. Cell counts (**B**) and total protein content (**C**) in BAL 24 h after CLP in WT and integrin β3^−/−^ mice. Serum (**D**) and BAL (**E**) cytokine IL-6 in mice alive at 0, 8,24 h were measured by ELISA. Data are expressed as mean ± SEM (n ≥ 3mice per group). *P < 0.05 versus control (0 h), ^#^P < 0.05versus WT CLP group. (**F**) Histopathologic analysis of lung from WT and β3^−/−^ mice 24 hours after CLP. Images(x20) from lung sections were shown (upper panel). Quantification of lung injury in H&E-stained lung tissue (bottom panel). The graph is representative of lung sections from three or more mice per group. *P < 0.05 versus control, ^#^p < 0.05 versus WT CLP group.

We recently reported that among the early events after CLP in mice was a significant increase in β6 integrin and WISP1 levels in the lung^10^. We now report a time dependent increase in integrin αv and β3 following CLP surgery. These increases were not observed in TLR4 and CD14 null mice (Fig. 2A). We subjected β3^−/−^ mice to CLP and analyzed the inflammatory cells numbers, protein content and IL-6 level in BAL fluid as well as in serum. At 24 h post CLP, lungs from β3^−/−^ mice had fewer inflammatory cells and lower protein content in BAL than WT mice (Fig. 2B,C). Furthermore, IL-6 levels in both the serum and BAL were significantly lower in β3^−/−^ mice (Fig. 2D,E). Histologic analysis of the lungs showed a reduction in the histologic changes induced by CLP in β3^−/−^ mice (Fig. 2F).

### Integrin αvβ3 is involved in WISP1 induced TNF-α release in RAW264.7 cells

**Figure 3:**
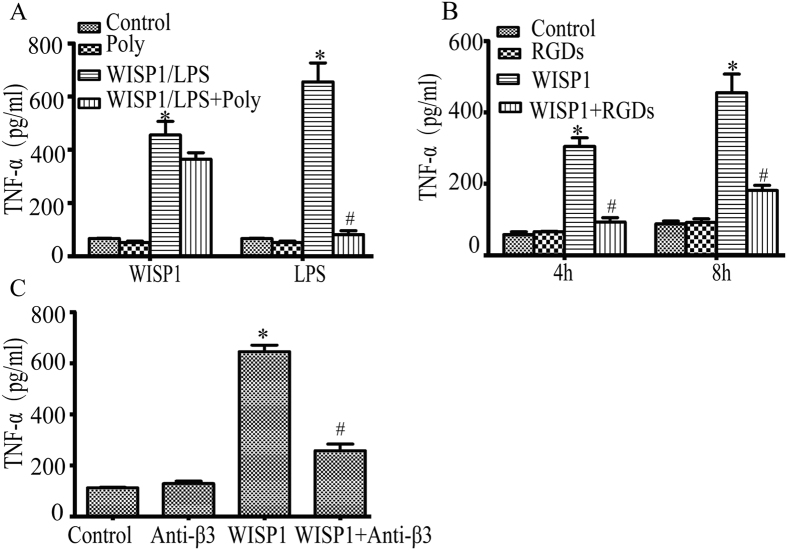
Integrin αvβ3 is involved in WISP1 induced TNF-α release in RAW264.7 cells. RAW cells were challenged with WISP1 (10 μg/ml) or LPS (10 ng/ml) in the presence or absence of polymyxin B for 8 h (**A**). RAW cells were pretreated with RGDs (10 μg/ml) (**B**) or anti- β3integrin antibody (10 μg/ml) (**C**) then stimulated with or without WISP1 for indicated time. TNF-α was analyzed by ELISA. Data are expressed as mean ± SEM of three independent experiments with similar results. *P < 0.05 versus control, ^#^p < 0.05 versus WISP1/LPS alone.

Although integrin αvβ3 is important for reducing vascular permeability changes in murine model of CLP^17^, there is nothing known of its role in the effects of WISP1 on macrophages –critical cell type in the context of sepsis. Exposure of RAW264.7 cells to recombinant WISP1 (10 μg/ml) promoted the release of TNF-α into culture medium at 8 h. The effect of recombinant WISP1 was not likely due to LPS contamination as it was unaffected by addition of polymixin B sulfate at a concentration (10 μg/ml) sufficient to abolish the response to exogenous LPS, itself (Fig. 3A). Integrins have been identified as signaling receptors mediating various CCN proteins functions. Inhibition of integrin signaling with RGD decreased WISP1 induced TNF-α production (Fig. 3B) suggesting an integrin dependent pathway. Integrin αvβ3 has been shown to be essential for TNF-α production in monocytic THP-1 cells^19^. To determine whether integrin αvβ3 contributes to WISP1-induced TNF-α release in Raw264.7 cells, we pretreated cells with an anti-β3 antibody (16-0611-81, eBioscience). Our results showed that blocking integrin αvβ3 by anti-β3 antibody (Fig. 3C) suppressed WISP1-induced TNF-α release. These observations demonstrate thatWISP1induced TNF-α release required integrin αvβ3.

### WISP1 synergistically enhances LPS induced TNF-α release, which is dependent on αvβ3 integrin receptors in PMø

**Figure 4:**
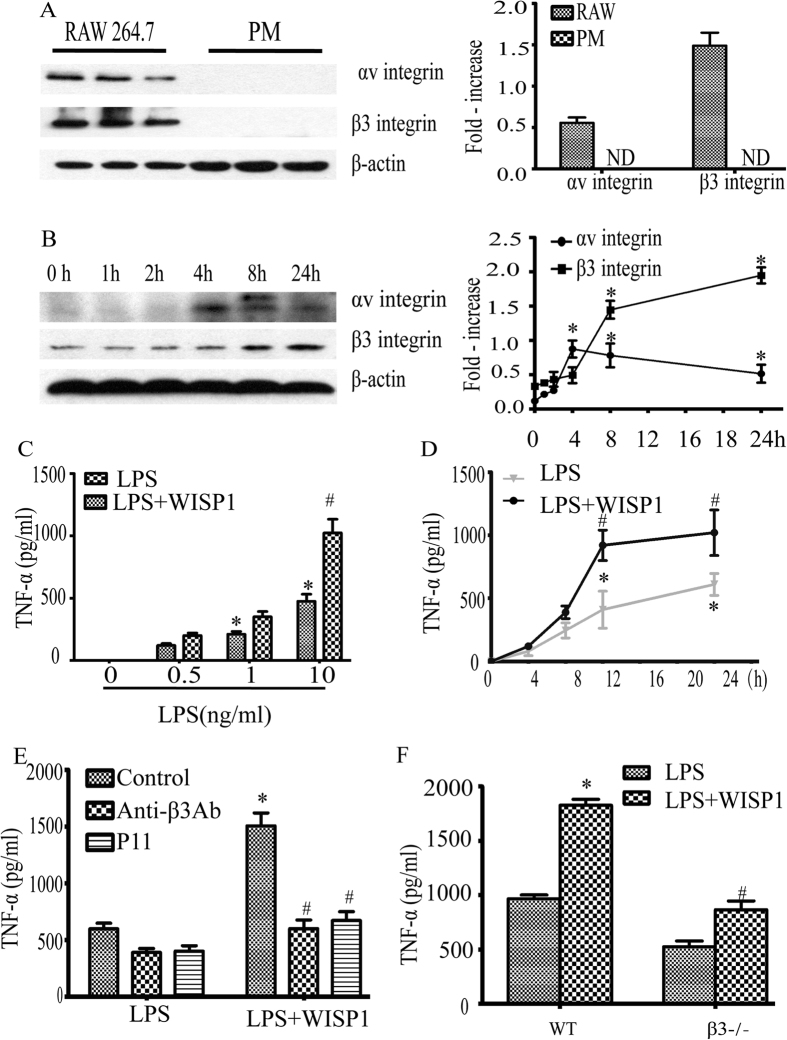
WISP1 synergically enhances LPS induced TNF-α release is dependent on the αvβ3 integrin receptors in PMø. Cell lysates from unstimulated RAW264.7 cells and PMø (**A**), and LPS (10 ng/ml) stimulated PMø (**B**) for indicated time were probed for αvβ3 integrin expression by western bloting (left panel) and analysis quantitative densitometry analysis of the protein expressions(right panel). The blots shown are representative of three experiments with similar results. *P < 0.05 versus 0 h; ND, none detected. Cells were co-incubation with WISP1 (10 μg/ml) and LPS at various concentration (0–10 ng/ml, **C**) or stimulation with LPS (10 ng/ml) in the presence or absence of WISP1 (10 ug/ml) for different time points (0–24 h, **D**). TNF-α was assessed using ELISA. Data are expressed as mean ± SEM of three independent experiments with similar results. *P < 0.05 versus control, ^#^P < 0.05 versus LPS (alone). Cells were pretreated with P11 (10 μg/ml) or anti-β3 integrin antibody (**E**) for 1 h, then stimulated with LPS (10 ng/ml) in the presence or absence of WISP1 (10 μg/ml) for 16 h. (**F**) PMø isolated from β3^−/−^ mice and WT mice were treated with LPS (10 ng/ml) in the presence or absence of WISP1 (10 μg/ml) at 16 h. TNF-α was analyzed by ELISA. Data are expressed as mean ± SEM of three independent experiments with similar results. *P < 0.05 versus LPS (alone), ^#^P < 0.05 versus LPS + WISP1.

WISP-1, by itself, did not increase TNF-α synthesis by PMø isolated from C57B/6 mice. We compared the integrin αvβ3 expression on Raw264.7 cells and PMø. We found that integrin αvβ3 expression was easily detectable at baseline in Raw264.7 cells but not in PMø (Fig. 4A). Previous studies have showed that αvβ3 integrin was highly expressed on activated cells under pathological conditions, and expression of αvβ3 integrin in monocytes can be up-regulated by stressful signals such as M-CSF and oxidized LDL^27^. To examine whether TLR4 agonist stimulation increased integrin expression in PMø, we treated cells with LPS and collected protein at different time points. Western Blot showed that LPS up-regulated αv and β3integrin in a time dependent manner (Fig. 4B).

Our previous study has shown that WISP1 was capable of enhancing TNF-α release in PMø^9^. In this study we confirmed that WISP1 synergistically enhanced LPS-induced TNF-α release in PMø. The enhanced effect mediated by WISP1 was more effective in 10 ng/ml of LPS (Fig. 4C). Using LPS at a concentration of 10 ng/ml, we noted that enhanced TNF-α production in the presence of WISP1 (10 μg/ml) was observed within 12 to 24 hrs following agonist stimulation (Fig. 4D).

As in RAW264.7 cells, once PMø were activated by LPS, the synergistic effect of WISP1 on TNF-α synthesis was partially dependent on αvβ3integrin by pretreating cells with anti-β3 antibody or an αvβ3 antagonist P11 (407272, Merck-Millipore) (Fig. 4E) suggesting a role for integrin receptors in the response. To confirm the importance of β3 integrin in the synergistic effects of LPS with WISP1 in macrophages, wild type or β3^−/−^ PMø were exposed to LPS in the presence or absence of WISP. The augmented of TNF-α release induced by WISP1 was marked reduced in the knockout cells. (Fig. 4F). These data together suggest that integrin αvβ3 is up-regulated in PMø under LPS stimulation and β3 is required for the response to WISP1. We also observed that block integrinβ3 impaired the response of LPS induced TNF-α release in PMø.

### TLR4 signaling dependent up-regulation of αvβ3 integrins

**Figure 5:**
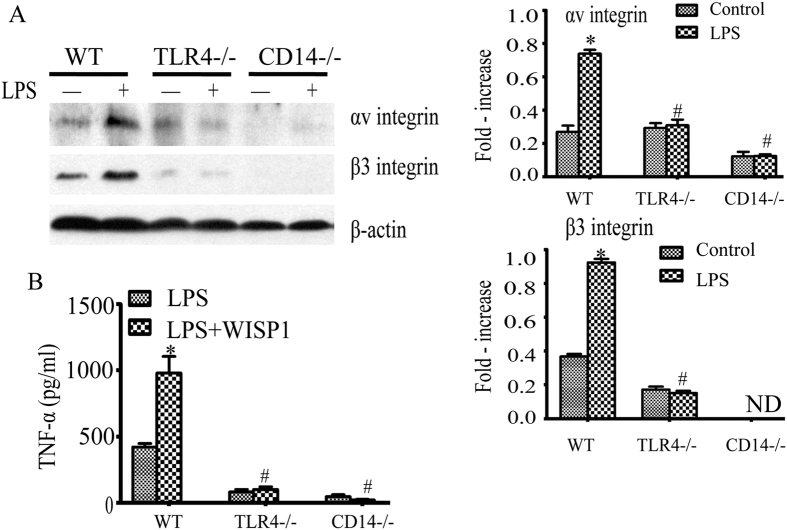
TLR4 signaling dependent up-regulation of αv and β3integrins. (**A**) PMø isolated from WT, TLR4^−/−^ and CD14^−/−^ were stimulated with LPS (10 ng/ml) for 8 h. Cell lysates were probed for αvβ3 integrin expression by western bloting (left panel) and quantitative densitometry analysis of the protein expressions (right panel). The blots shown are representative of three experiments with similar results. *P < 0.05 versus control, ^#^P < 0.05 versus WT LPS. (**B**) Cells incubated with LPS (10 ng/ml) with or without WISP1 for 24 h and TNF-α was analyzed by ELISA. Data are expressed as mean ± SEM of three independent experiments with similar results. *P < 0.05 versus LPS, ^#^P < 0.05 versus LPS + WISP1.

To determine if canonical TLR4 signaling is involved in the up-regulation of αvβ3 integrin, PMø isolated from wild type, TLR4^−/−^ and CD14^−/−^ mice were exposed to LPS. As shown in Fig. 5A, αv and β3 expression were increased following exposure to LPS in the wild type cells but absent in TLR4^−/−^ and CD14^−/−^ PMø stimulated with LPS. To answer if the changes in αvβ3 expression correlated with TNF-α production, cells from these same strains were exposed to LPS with or without WISP1. We confirmed a suppression in TNF-α production in TLR4^−/−^ and CD14^−/−^ PMø in response to LPS in the presence of WISP1 (Fig. 5B). These data indicate that the synergistic effect of WISP1 with LPS is also dependent on TLR4/CD14.

### ERK activation links WISP1 increased in TNF-α production and αvβ3 integrin expression by PMø exposed to LPS

**Figure 6:**
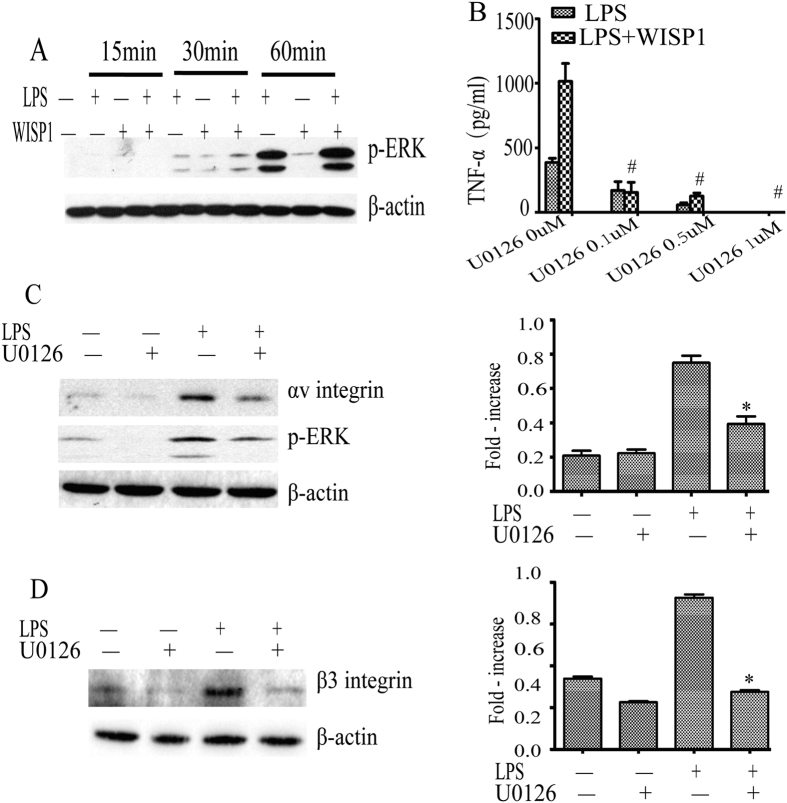
ERK activation links WISP1 increased in TNF-α production and αvβ3 integrin expression by PMø exposed to LPS. (**A**) PMø were incubated with LPS in the presence or absence of WISP1 for indicated time interval, and p-ERK was examined by western blot analysis. (**B**) PMø were pretreated with ERK inhibitor (0–1 μM) for 1 h then stimulated with WISP1 and LPS for 24 h and TNF-α was analyzed by ELISA. Data are expressed as mean ± SEM of three independent experiments. ^#^P < 0.01 versus without U0126 pretreatment. PMø were pretreated with U0126 (0.5 μM) for 1 h then stimulated with LPS for 8 h (**C**) or 24 h (**D**) and detected αv and β3 integrin protein expression by Western blot. *P < 0.05 versus LPS (alone).

WISP1 signaling was shown to have a role in chondrosarcoma cells and promotes cell mobility through ERK signaling^28^. To test whether ERK involved in WISP1-mediated augmentation of TLR signaling, we examined the phosphorylation status of ERK in cells exposed to LPS with/without WISP1. As shown in Fig. 6A, there was a strong additive effect between LPS and WISP1for ERK phosphorylation. To determine if ERK activation was involved in TNF-a production in response to LPS, or WISP1, we treated cells with the ERK inhibitor U0126. As shown in Fig. 6B, U0126 indeed suppressed TNF-α production in a dose dependent manner. The synergistic effect of WISP1 was abolished in the absence of ERK signaling. These results suggest that ERK activation links WISP1 enhanced effect on TNF-α production on macrophages.

To examine the role of ERK in LPS regulated αv and β3 integrin expression, we pretreated PMø with U0126, then stimulated with LPS and detected integrin protein expression. As shown in Fig. 6C, the level of ERK phosphorylation decreased at 4 hours after exposing the macrophage to 0.5 μM U0126. Inactivation of ERK accompanied the decrease of αv integrin in the presence of LPS. Further, pretreatment with U0126 significantly blocked the up-regulation effect of β3 integrin by LPS at 24 h post treatment (Fig. 6D). This result is consistent with the observation of ERK regulating the synergistic effect by WISP1 and LPS on TNF-α production.

## Discussion

WISP1 is a member of the cysteine-rich **CCN** protein family of growth factors that includes **C**ysteine-rich protein 61 (Cyr61), **C**onnective tissue growth factor (CTGF), and **N**ephroblastoma over-expressed protein (*Nov*). In our recent study, we found that intrapulmonary WISP1 is elevated in polymicrobial sepsis^10^. This adds to previous findings that WISP1 increases after sterile lung injury with bleomycin^7^ or high tidal mechanical ventilation^9^.The induction of WISP1 in ventilator induced lung injury (VILI) was dependent upon TLR4 and WISP1 was shown to co-immunoprecipitated with the functionally active glycosylated form of TLR4. Furthermore, WISP1 appeared to be an accessory molecule facilitating TLR4 mediated TNF-α synthesis in LPS treated peritoneal macrophages^9^. Although WISP1 has been reported to be important in repair of respiratory epithelium^28^, in the context of the injuries noted above, it appears to contribute to lung inflammation and pathology suggesting that its role is dependent upon the nature of the stimulus as well as the cellular and integrated pulmonary response^29^.

Previously studies indicated that at least eight integrins (αvβ3, α2β1, α5β1, α6β1, αvβ5, αIIbβ3, αMβ2 and αDβ2) can serve as signaling receptors mediating CCN functions^13^. In the current study, we show that WISP1 and integrin αvβ3 are elevated in lungs of mice after CLP and the increase in integrin αvβ3 was dependent upon TLR4 and CD14. We also observed that integrin αvβ3 expression were increased following exposure to LPS in the wild type cells but absent in TLR4^−/−^ and CD14^−/−^ PMø under LPS stimulation. Membrane-bound integrin β3 were shown to be key players in cancer metastasis^30^. We test integrin β3 on cell surface by flow cytometry, but we did not observe LPS increased integrin β3 on cell surface (data not shown) as well as expression. Integrin diversity and function is regulated by alternative splicing. Younis Skaik *et al*.^31^ demonstrated that secreted integrin (sβ3) has a immunomodulatory functions including induces the secretion of pro-inflammatory cytokines in Natural Killer Cells. We speculated sβ3 may be involved in WISP1’s synergistic effect. Indeed, which forms of integrinβ3 are increased by LPS stimulation and strongly associated with WISP1’s synergistic effect in PMø need further study to be conclusive.

The RGD sensitive nature of WISP1 mediated lung injury in CLP^10^ encouraged us to pursue a role for integrins in the pro-inflammatory state of the lungs in sepsis and thus we focused on cell culture models of TNF-α release from macrophages. The lack of response of primary cultures of PMø to WISP1 alone, in contrast to a robust response in RAW264.7 cells (Fig. 3A) led to a survey of candidate integrins which may be involved in WISP1 induced TNF-α release. Antonov *et al*^18^. demonstrated that αvβ3 integrin ligation resulted in NF-κB activation and increasese in pro-inflammatory cytokines mRNA expression and secretion. We hypothesize that integrin αvβ3 contributes to WISP1 induced TNF-α secretion. We found that RAW264.7 cells expressed easily detectable levels of both proteins at the baseline, whereas PMø did not. Blocking integrin αvβ3 by an antibody inhibited WISP1 induced TNF-α release in RAW264.7 cells. Integrin αvβ3 was inducible in PMø with TLR4 agonists and may enable WISP1 to further increase TNF-α release from PMø. The loss of this effect in experiments repeated in PMø isolated from β3 null mice suggested the requirement of integrinβ3 (Fig. 3D). Gene knockout mice allow individual molecules in the pathology to be assessed, but the overall importance of findings is occasionally confounded by enforced compensatory mechanisms. Lerman YV *et al*.^32^ found that α_3_β_1_ deficiency promotes compensatory CD11b up-regulation and impacts TLR2 signal transduction. Strong other experimental support has been provided for the injurious role of integrin β5^33^,^34^ and β6^35^ on pulmonary endothelium and epithelium in ALI^12^. Indeed, we reported that neutralizing antibodies to integrin β6 reduced ALI after CLP and also reduced the up-regulation of WISP1. So we can not exclude the possibility that other integrins may be involved in this effect. We also observed that blocking integrin β3 by P11 pretreatment or using β3 null macrophages impaired the response of LPS for TNF-α release *in vivo*. The effect and mechanism of αvβ3 integrin regulated macrophage inflammation under LPS stimulation has been demonstrated via PI3 kinase/Akt-dependent NF-κB activation^18^. Therefore, the role of integrin β3 in regulating the response of TLR4 could extend beyond the reposes to WISP1.

Macrophage activation is a key step in the pathophysiology of sepsis-associated tissue injury. Our experiments using PMø isolated from TLR4 and CD14 null mice provided support for the notion that a TLR4-integrinβ3-WISP1 complex or interaction underlies WISP1 effects on TNF-α release from macrophages. Ablation of integrin β3 is protective in sepsis induced lung injury (Fig. 2). Interestingly, a recent study by Su G and colleagues^17^ using integrin β3 knockout mice demonstrated that theses knockout mice have increased vascular leak and pulmonary edema formation after endotracheal LPS, and increased vascular leak and mortality after intraperitoneal LPS and CLP than WT mice. The difference between the results presented here and those reported by Su G *et al*.^17^ may be due to a number of factors. The model applied by Su G *et al*. produced a sub-lethal CLP model in the WT mice by 250 hs post-CLP, whereas our model produced lethal CLP mode with 80% mortality in the wild-type mice by 3 days post-CLP (Fig. 1A). It has been previously shown that the mortality rate and immune response is highly dependent on the severity of CLP. For example, José C and colleagues^36^ demonstrated that TLR4 signaling is not essential in sub-lethal polymicrobial sepsis induced by both CLP and polymicrobial inoculation models, but it is crucial in lethal polymicrobial sepsis, since TLR4-deficient mice that underwent lethal CLP or polymicrobial inoculation presented low bacteremia and a high survival rate and did not display systemic inflammation. Therefore the mild model of CLP is most likely distinct from our model. With this more severe model we were see a difference in sepsis induce lung injury between Integrin β3^−/−^ and WT mice. A second factor is that Su G *et al*. used female mice whereas our study examined males. Numerous studies have documented the divergent effects of gender on mortality subsequent to traumatic injury/shock and/or sepsis^37^. Nonetheless, this might be another explanation for some of the differences between Su G *et al*. and our study.

In conclusion, our findings demonstrated for the first time, that signaling initiated by WISP1-integrin ligation modulated LPS induced ERK activation and primed macrophages to an enhanced response to LPS exposure. Signaling through TLR4, resulted in a rapid up-regulation of both αv and β3 integrin in macrophages. We thus postulate that WISP1-Integrin signaling interacts with TLR4 and/or their signaling pathways and results in enhanced LPS induced cytokine secretion. Furthermore, TLR4 signaling up-regulate integrins expression that forms a positive loop for cytokine release. These data underscore the contribution of a TLR4-αvβ3 dependent, WISP1 mediated release of the inflammatory mediator in macrophages and suggest a novel pathway that may be therapeutically modified by a variety of small molecules and/or neutralizing antibodies in the lung to both infectious and sterile injury.

## Material and Methods

### Animal

Male C57BL/6, TLR4^−/−^, CD14^−/−^ were bred at the core facility at the University of Pittsburgh and integrin β3^−/−^ (mutant) purchased from Jackson laboratory. All mice were used at the age of 8–12 wk. All mice were developed on a C57BL/6 genetic background. Animal protocols were approved by the Animal Care and Use Committee of the University of Pittsburgh, and the experiments were performed in strict adherence to the National Institutes of Health Guidelines for the Use of Laboratory Animals.

### Murine Model

The CLP murine model was used to induce fecal peritonitis. In brief, mice were anesthetized with isoflurane, and a middle abdominal incision was made. The cecum was mobilized, ligated, and punctured through-and-through with a 20-gauge needle, allowing exposure of faces, the bowel was repositioned, the abdomen was closed and sterile saline-solution was administered for fluid resuscitation. In sham group mice, the cecum was exposed and the bowel was massaged as described above, except that the cecum was not ligated or punctured. At the end of the experiment, left lungs were snap frozen in liquid nitrogen for subsequent biochemical analysis. Right lungs were fixed with 4% buffered formalin for histological examination. The degree of lung injury was calculated based on the histologic scoring system described by Oishi H *et al*.^38^. In separate experiments, BAL were assessed for total cell counts and protein concentration and interleukin 6 (IL-6) as well as serum.

### Cell culture

PMø were isolated from C57BL/6 or the indicated gene knock out mice, 3d after i.p. injection of 2 ml 4% thioglycollate and plated in RPMI1640 medium containing 10% FBS, 50 U/ml penicillin G sodium and streptomycin sulfate and maintained overnight including washing away floaters with PBS and incubated in fresh medium. On the next day, the adherent macrophages were incubated in serum-free medium RPMI1640 at 37 °C for 2 h and then stimulated with LPS(List Biological Laboratories, *Escherichia coli* 055:B5),WISP1 (R&DSystems, 1680-ws-050), or their combination. RAW264.7 cell line was obtained from American Type culture collection that was originally established from a tumor, induced by Abelson leukemia virus and cultured in DMEM containing 10% FBS at 37 °C with 5% CO_2_.

### ELISA

Cell culture medium and serum were collected after indicated stimulation or surgical procedures. Measurement of TNF-α or IL-6 concentration were carried out using TNF-α /IL-6 ELISA kit (R&D Systems) following the manufacturer’s protocol.

### Western Blotting

Anti- integrin αv antibody was obtained from Millipore (AB1930, 1:1000), anti- integrinβ3 antibody from Abcam (ab75872, 1:1000). Anti-phosphorylated ERK was obtained from Cell Signaling Technology (#9101, 1:1000). Immunoblot analysis was performed as described previously^10^. Tissue or Cells were lysed in cell lysis buffer (Cell Signaling) with protease inhibitor cocktails (Sigma). Lysates were centrifuged at 15,000 *g* for 15 min at 4 °C, and supernatants were collected. 20–30 μg of cell lysates were separated by SDS-PAGE gel and transferred to Reinforced NC membrane (Whatman GmbH). The membranes were blocked with 5% skim milk in 0.1% Tween 20/TBS buffer and then incubated with primary antibodies. Blots were probed with horse radish peroxidase (HRP)-conjugated anti-mouse or anti-rabbit IgG (Jackson ImmunoResearch). Bands were visualized using Super Signal West Pico Chemiluminescent Substrate (Thermo Scientific).

### Statistical Analyses

All results are expressed as means ± standard error of the mean (SEM) for data resulting from *in vivo* and *in vitro* analyses. Statistical analysis was performed by one-way ANOVA followed by Tukey’s multiple comparison test. P < 0.05 was considered statistically significant.

## Additional Information

**How to cite this article**: Chen, Z. *et al*. WISP1-αvβ3 integrin signaling positively regulates TLR-triggered inflammation response in sepsis induced lung injury. *Sci. Rep.* **6**, 28841; doi: 10.1038/srep28841 (2016).
